# Exploring New Kingdoms: The Role of Extracellular Vesicles in Oxi-Inflamm-Aging Related to Cardiorenal Syndrome

**DOI:** 10.3390/antiox11010078

**Published:** 2021-12-29

**Authors:** Cristina Mas-Bargues, Matilde Alique, María Teresa Barrús-Ortiz, Consuelo Borrás, Raquel Rodrigues-Díez

**Affiliations:** 1 Grupo de Investigación Freshage, Departmento de Fisiología, Facultad de Medicina, Universidad de Valencia, 46010 Valencia, Spain; cristina.mas@uv.es (C.M.-B.); Consuelo.borras@uv.es (C.B.); 2 Instituto Sanitario de Investigación INCLIVA, 46010 Valencia, Spain; 3 Centro de Investigación Biomédica en Red Fragilidad y Envejecimiento Saludable, Instituto de Salud Carlos III (CIBERFES, ISCIII), 28029 Madrid, Spain; 4 Departamento de Biología de Sistemas, Universidad de Alcalá, 28871 Madrid, Spain; matilde.alique@uah.es; 5 Instituto Ramón y Cajal de Investigación Sanitaria (IRYCIS), 28034 Madrid, Spain; 6 Área de Fisiología, Departamento de Ciencias Básicas de la Salud, Facultad de Ciencias de la Salud, Univesidad Rey Juan Carlos, Avenida de Atenas s/n, 28922 Madrid, Spain; 7 Departamento de Farmacología, Facultad de Medicina, Universidad Autónoma de Madrid, 28029 Madrid, Spain; Raquel.rodrigues@uam.es; 8 Instituto de Investigación Hospital La Paz (IdiPAZ), 28046 Madrid, Spain; 9 Centro de Investigación Biomédica en Red en Enfermedades Cardiovasculares (CIBERCV), 08036 Barcelona, Spain

**Keywords:** aging, oxidative stress, senescence, extracellular vesicles, senolytics, inflammation, age-related pathologies, oxi-inflamm-aging

## Abstract

The incidence of age associated chronic diseases has increased in recent years. Although several diverse causes produce these phenomena, abundant evidence shows that oxidative stress plays a central role. In recent years, numerous studies have focused on elucidating the role of oxidative stress in the development and progression of both aging and chronic diseases, opening the door to the discovery of new underlying mechanisms and signaling pathways. Among them, senolytics and senomorphics, and extracellular vesicles offer new therapeutic strategies to slow the development of aging and its associated chronic diseases by decreasing oxidative stress. In this review, we aim to discuss the role of extracellular vesicles in human cardiorenal syndrome development and their possible role as biomarkers, targets, or vehicles of drugs to treat this syndrome.

## 1. Introduction

In 1956, Harman et al. [1] proposed the “free radical theory of aging”, where the authors described how oxidative stress generates cell damage associated with aging. Another well-established theory is that of the “Hayflick limit”, which states that every cell has a maximum limit of divisions [2]. Both theories are related to each other, as ROS accumulation can reduce the Hayflick limit, thereby shortening the life span. This phenomenon is known as premature senescence due to oxidative stress. Moreover, senescent cells release several bioactive molecules, termed senescence-associated-secretory phenotypes (SASP), creating a pro-inflammatory ambiance. Indeed, the SASP may contribute to chronic inflammation, often referred to as inflammaging [3].

Each of these features, oxidative stress, senescence, and chronic inflammation, lead to the appearance of age-related diseases.

### 1.1. Aging and Oxidative Stress

Aging can be defined as the progressive loss of tissue and organ function over time [4]. Age-related functional losses are due to the accumulation of reactive oxygen and nitrogen species (RONS) that damage lipids, DNA, proteins, and carbohydrates [1,5]. Cells have developed RONS scavengers to maintain cellular homeostasis, i.e., an antioxidant defense system that includes superoxide dismutase (SOD), catalase, and glutathione peroxidase (GPx).

The balance between oxidants and antioxidant systems in the aging process seems to show a trend for oxidative stress [6]; however, this is not always true. Several studies did not find a positive correlation between oxidative stress and age when comparing healthy elderly people [7,8]. Indeed, a systematic review that analyzed the centenarian cohort, who represent a model of successful aging, concluded that these long-lived individuals present less oxidative damage and significantly lower plasma lipid peroxidation biomarkers than control individuals [9]. Thus, oxidative damage does not correlate with age, especially in the geriatric population, but rather with the frailty state, where frailty is a multifaceted geriatric disorder characterized by lower physical activity, decreased muscle strength and endurance, and the inability to cope with stress.

### 1.2. Aging and Senescence

Cellular senescence can be described as a stable state of growth arrest in which cells are unable to proliferate in response to several stresses. Senescent cells are characterized by a flattened and enlarged morphology, increased expression of cell cycle-inhibitory proteins such as p16^INK4a^, and higher senescence-associated β-galactosidase activity. Senescent cells exert their pleiotropic biological functions through the transcriptional activation of a SASP program composed of cytokines, chemokines, growth factors, extracellular matrix proteases, and even extracellular vesicles (EVs) that affect the local tissue microenvironment [10]. Indeed, cellular senescence entails chronic inflammation through the SASP and impairs tissue regenerative potential when affecting stem cells, leading to chronic age-associated diseases and organismal aging [11].

Moreover, cellular senescence has also been detected in immune cells, driving a declining immune system function, a phenomenon called immunosenescence. This phenomenon leads to the impaired clearance of senescent cells, thereby contributing to their accumulation in the tissues. Cellular senescence accumulates systematically over time, affecting both mitotic and postmitotic cells and spreading SASP factors, thus leading to tissue dysfunction and pathology [12].

### 1.3. Aging and Inflammation

With advancing age, individuals tend to develop a chronic pro-inflammatory condition, characterized by high circulating levels of inflammatory molecules, known as “inflammaging” [13]. Inflammaging describes the low-grade, chronic, systemic inflammation in aging and is a highly significant risk factor for both morbidity and mortality in older people.

As previously mentioned, the accumulation of senescent cells and their associated pro-inflammatory secretome is a constant trigger of inflammaging [14,15]. The level of cytokines often remains within the upper limit of the normal range; several mediators secreted by monocyte/macrophages such as tumor necrosis factor-alpha (TNFα), interleukin-1 (IL-1), and interleukin-6 (IL-6), as well as chemokines such as monocyte chemoattractant protein-1 (MCP-1) and interleukin-8 (IL-8), are increased. On the other hand, anti-inflammatory mediators such as IL-10, IL-4, and IL-13 may also be increased as a tentative measure to control this state [16]. Nonetheless, long-lived people, such as centenarians, can cope with chronic inflammation through an anti-inflammatory response called “anti-inflammaging” [17].

### 1.4. Extracellular Vesicles and Aging

In the 1980s, a new cell–cell communication system was discovered based on the action of vesicles that carry bioactive molecules to neighboring cells [18]. These vesicles, known as EVs, act as regulators of several pathophysiological processes and participate in the development and progression of multiple diseases [19]. EVs contain specific proteins, lipids, and nucleic acids (including DNA, RNA, and miRNA) that mirror parental cells, and can report active metabolites from their cell or tissue of origin to neighboring cells [20,21,22].

In general, three EVs types have been described depending on the size, morphology, biochemical composition, and release mechanism [23]. However, due to the lack of unique markers for defining the different kinds of EVs and their heterogeneous size, small or large EVs [24], there is a discrepancy in the nomenclature (Table 1). Therefore, in this review, we have generally referred to EVs.

EVs can mediate beneficial effects [25]. However, elevated plasma levels of EVs are involved in the etiopathogenesis of many chronic inflammatory diseases. They have been detected in patients with cardiovascular risk factors (diabetes, hypertension, peripheral vascular disease, heart failure), aged people, and individuals who have suffered from CKD and cancer [25,26,27]. Thus, EVs are emerging as promising candidates in clinical diagnosis and a possible alternative in monitoring the therapeutic follow-up, acting as biomarkers due to their involvement in developing senescence in chronic inflammatory pathologies, such as CVD-associated-CKD [26,28].

## 2. Cardiovascular Diseases as the Paradigm of Age-Related Chronic Diseases: Cardiorenal Syndrome

Age is a risk factor for CVD and chronic kidney diseases (CKDs) [29]. The prevalence of CVD in the elderly (those over 65 years old), especially in those over 80 years of age, is over 80% [30], whereas CKD prevalence is around 50% in patients who are 70 years old or older [31]. Furthermore, both are expected to increase by 10% over the next 20 years due to the growth of the elderly population and the increase of other risk factors such as diabetes mellitus, obesity, or hypertension [30,32]. Thus, both pathologies have become a significant threat to public health in modern societies [33,34].

A remarkable fact is that kidney failure is also a major cause of cardiovascular morbidity and mortality [32]. Indeed, epidemiological studies have demonstrated that CKD is a significant risk for cardiovascular events independently of classical risk [35,36]. Moreover, several studies have pointed out that CKD patients undergo accelerated aging, which enhances the appearance of CVDs [37]. Thus, although CKD and CVD share classical Framingham risks factors [38], this fact does not fully explain the uneven increase in cardiovascular morbidity and mortality in CKD patients. This has led many research groups to look for new mechanisms explaining the high cardiovascular syndrome presented in CKD patients. As a result, a new term has arisen: cardiorenal syndrome (CRS), which suggests the existence of common mechanisms and mediators involved in CVD and CKD progression [39]. However, this link is complex and remains poorly understood; therefore, the physio-pathogenic mechanisms by which CKD increases the risk of cardiovascular events are currently under intensive investigation. Several studies suggest that various substances accumulated in the bloodstream during CKD, such as uremic toxins, pro-inflammatory chemokines and cytokines, reactive oxygen species, and extracellular vesicles, come into direct contact with the endothelial cells causing endothelial damage and dysfunction, vascular inflammation, and oxidative stress and, consequently, vascular remodeling, thus triggering the onset of CVD [40].

### 2.1. Age-Related Changes in Renal and Cardiovascular System

**Figure 1:**
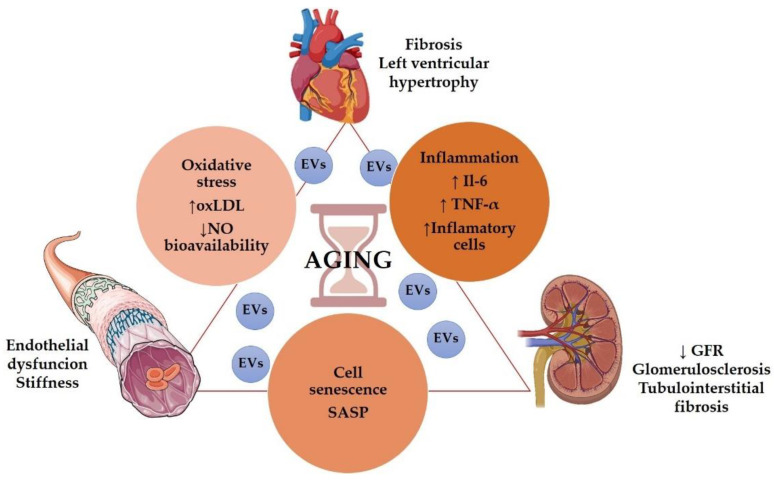
Role of aging in the cardiorenal syndrome. Some graphical elements from this figure were adapted from BioRender (http://biorender.com, accessed on 1 December 2021) and the Servier Medical ART (SMART) Powerpoint image bank (http://smart.servier.com, accessed on 1 December 2021).

Even in the absence of other risk factors, the aging population presented structural and functional alterations in the kidneys, vessels, and heart. (Figure 1).

Age is associated with a decrease in renal function [41], including glomerular filtration rate (GFR) decline and impaired urine concentrating capacity [42]. Even without any injury, GFR declined approximately 8 mL/min/1.73 m^2^ per decade after 40 years of age [43], but it has been suggested that GFR decline may start even earlier in the patient’s 20s [44]. Structurally, the aging kidney presented glomerulosclerosis, tubulointerstitial fibrosis, and tubular atrophy [45]. Besides, heart failure is a condition classically related to the elderly [46]. The most common pathophysiological characteristics of an aging heart are increased left ventricular (LV) hypertrophy and fibrosis [47]. Elderly patients presented diastolic dysfunction, increased atrial fibrillation, and a reduction in cardiac reserve [48]. Regarding vascular aging, it is characterized by endothelial dysfunction, mainly due to decreased nitric oxide (NO) availability [49], large arteries walls thickening and progressive stiffness of central arteries, particularly the aorta [48,50], resulting in atherosclerosis Figure 1.

Several mechanisms may participate in aging-induced cardiovascular and renal structural and functional impairments, including cell senescence, inflammation, oxidative stress, and genetic and epigenetic modifications.

### 2.2. Cellular Senescence in Cardiovascular and Renal Aging

Cellular senescence is a process characterized by a stable cell–cycle arrest [51] that causes inflammation and the capacity to modify the microenvironment through the SASP. The accumulation of senescent cells in the kidney, heart, and vascular vessels has been associated with structural and functional changes related to aging [52]. Furthermore, the acquisition of a senescent phenotype by aging or age-related chronic disease, including CKD and CKD-associated CVD, seems to be an irreversible pathophysiological process [22,53].

In large vessels, aging-associated endothelial cell senescence is a key cause of vascular structural changes and vascular dysfunction observed in atherosclerosis [45]. Additionally, human atherosclerotic plaque vulnerability is promoted by senescence vascular smooth muscle cells (VSMCs) [54]. Further osteoblastic-like phenotypes acquired by senescent VSMC seem to be responsible for vascular calcification [55]. In the kidney, the source of senescent cells depends on the pathology, and proximal tubular cells are the main source of senescent cells in the aged kidneys.

### 2.3. Inflammation in Cardiovascular and Renal Aging

Inflammaging describes the pro-inflammatory state observed in the older organism, even in the absence of other risk factors or diseases [13]. Several epidemiological studies have pointed to inflammaging as a risk factor of most age-related diseases, including CVD and CKD [56,57]. Chronic inflammation is a key factor in several CVD pathologies [58] and a key contributor to CKD development and its progression to end-stage renal disease (ESRD) [59]. Moreover, proinflammatory cytokines and chemokines released by kidneys can reach the circulation, resulting in dysfunction of distant organs, including the cardiovascular system [60], a fact that may explain, at least in part, the accelerated cardiovascular aging observed in CKD patients [37].

Among the inflammatory mediators elevated in blood during aging, IL-6 and TNF-α are particularly noteworthy [61]. An elevation of both IL-6 and TNF-α and other molecules such as C-reactive proteins have been associated with high mortality in the elderly [62]. Even in centenarians, elevated levels of TNF-α correlated with morbidity, including CVD and mortality [63]. On the other hand, both molecules are also key factors in the onset and development of renal and CVDs [64,65,66,67]. Indeed, both molecules are considered uremic toxins and are therefore molecular markers and/or therapeutic targets for cardiorenal syndrome.

Elevated IL-6 and TNF-α levels have been observed in CKD patients, and these levels are inversely correlated with GFR [68]. Moreover, elevated IL-6 levels and TNF-α have been associated with the development of atherosclerosis and vascular calcification in CKD patients [69,70,71]. Likewise, IL-6 has been proposed as a risk factor for left ventricular hypertrophy in peritoneal dialysis patients [72].

The human GG polymorphism at the −174 position in the promoter region of the IL-6 gene, which is associated with increased levels of IL-6, has been related to an increased risk of developing age-associated CVD [73,74] and with increased mortality in peritoneal dialysis patients [75]. On the other hand, this polymorphism is less frequent in centenarians than in young adults [76], whereas other IL-6 SNPs have been associated with longevity [77,78]. Moreover, in aged patients, including centenarians, high levels of TNF-α in the blood were associated with a high prevalence of atherosclerosis [63,79].

### 2.4. Oxidative Stress in Cardiovascular and Renal Aging

As previously indicated, the oxidative stress theory of aging states that age-associated loss of functionality would be due to the accumulation of oxidative damage to lipids, DNA, and proteins by RONS [5]. However, recent studies have demonstrated a more complex relation between oxidant and antioxidant mechanisms in aging and age-related diseases [80].

Oxidative stress is a key component of several age-related pathologies, including CVDs and acute CKD, and the role of different pro-oxidant molecules, as well as the therapeutic effects of several antioxidants, have been widely studied both in experimental models and in clinical trials [81,82,83,84,85,86,87,88].

CKD and ESRD patients show increased levels of different oxidative stress markers, including advanced oxidation protein products, malondialdehyde, and oxidized-low density lipoproteins (ox-LDL), which have been associated with a decline in renal function. Furthermore, an increase in ox-LDL, together with high IL-6 levels, has been associated with an increased risk of CVD events and CVD-related mortality in CKD patients in hemodialysis (HD) [89] and accelerated atherosclerosis development observed in CKD [82]. Besides its role in foam cells formation within the arterial wall, ox-LDLs also participate in other proatherogenic events, including endothelial dysfunction and smooth muscle proliferation, suggesting an essential role of ox-LDLs in atherosclerotic plaque development and destabilization [90,91].

Furthermore, increased levels of ox-LDL in older adults have also been associated with arterial stiffening [92]. However, another study in aged patients reported no correlation between ox-LDL levels and cardiovascular morbidity nor mortality, suggesting that in elderly patients, the ox-LDL may not be a good marker [93]. What seems clear is that ox-LDL levels are related to endothelial dysfunction observed in adults and elderly individuals [49].

Oxidative stress induces endothelial dysfunction by decreasing NO bioavailability [94], mainly by the formation of peroxynitrite (ONOO^−^), through its combination with superoxide anion (O_2_^•−^), which is elevated in atherosclerotic lesions [95]. Moreover, ONOO^−^ also leads to endothelial nitric oxide synthase (eNOS) uncoupling activity, thus perpetuating the detrimental response. In addition to its role in endothelial function, NO has other effects, including antithrombotic, anti-inflammatory, and anti-atherogenic effects [49]. Therefore, in vascular endothelium, ox-LDL and NO exert antagonistic actions in all phases of atherogenesis. Indeed, some authors have proposed using ox-LDL to NO ratio (ox-LDL/NO) as a new biomarker for endothelial dysfunction in atherosclerosis [49]. Curiously, whereas NO produced by eNOS seems to have atheroprotective effects, excessive NO produced by inducible nitric oxide synthase (iNOS), under proinflammatory conditions, had detrimental effects in the endothelium [95]. Conversely, elderly humans presented elevated NO production within the vasculature but a reduced NO bioavailability. In the kidney, aging-associated NO reduction increases renal vascular vasoconstriction, Na^+^ retention, and renal fibrosis, thus contributing to enhanced hypertension and declined renal function [45].

Finally, given the close relationship between oxidative stress, inflammation, and aging, the free radical theory of aging has been updated, giving rise to the oxidation-inflammatory theory of aging or oxi-inflamm-aging [96]. This new theory postulates that aging is a loss of body homeostasis due to sustained oxidative stress that activates different systems, including the immune system, thus inducing an inflammatory response that increases oxidative stress and perpetuating positive feedback of oxidative stress and inflammation.

### 2.5. Extracellular Vesicles in Cardiovascular and Renal Aging

In human renal and cardiovascular pathologies, changes in composition and levels of EVs have been described [97]. In addition, different studies showed the effect of drug treatment on EVs’ profile in different diseases [98]. Altogether these results point to a potential role of EVs as biomarkers for diagnosis and as tools for therapy by drug administration of different cargo.

In CKD, circulating EVs are augmented and are key players in vascular calcification [99], endothelial dysfunction [100], and vascular mortality [101]. In hemodialyzed patients with CKD, plasma circulating EVs were increased compared with elderly subjects without CKD used as controls [102]. In this study, the level of EVs released by proinflammatory monocytes was high, and no differences in total monocyte-derived EVs were found as other authors had previously described [103,104,105]. The uremic toxin proinflammatory environment in these CKD patients induces proinflammatory monocytes activation, alters miR-126-3p, miR-233-3p, miR-192-5p expression, and increases the release of proinflammatory EVs that enhance vascular inflammation. As miR-126-3p participates in endothelial proliferation and endothelization in large vessels [106,107,108], the decreased miR-126-3p circulating levels reported in these hemodialyzed patients indicate its implication in the vascular dysfunction observed [102].

In addition, the decrease in miRNA-233-3p expression and circulating levels observed in CDK patients was reversed and even increased after kidney transplantation [109,110], indicating its participation in vascular complications development. The lower expression of miR-192-5p was also found in hemodialyzed patients [102], venous thromboembolism [111], and hypertension [112]. As the expression of several miRNAs can be positively or negatively correlated with different diseases and inflammatory states, authors consider those miRNA ratios to be a clinical feature of every disease and a diagnostic and therapeutic biomarker.

The mentioned studies suggest that serum levels and the profile of miRNAs and EVs depend on CKD’s uremic inflammatory state and promote cardiovascular damage [102].

The progressive decrease in renal function is a risk factor in most CVDs and worsens the clinical outcomes [113,114].

For example, non-valvular atrial fibrillation is linked to kidney disease because of increased thromboembolism mediated by higher levels of EVs from the prothrombotic endothelial-platelet origin but not by other markers of thrombotic state and cellular activation [115] even in anticoagulated patients.

In hypertensive patients, the presence of EVs indicating podocyte injury, a characteristic expression of miRNAs, and peritubular capillaries damage has been described [116]. Furthermore, EVs released by endothelial cells from perivascular capillaries had been detected in the urine of essential and renovascular hypertensive patients, the concentration of which directly correlates with clinical parameters and capillary rarefaction but inversely with renal perfusion [117]. Therefore, the levels of urinary EVs in hypertension could be an early marker of renal injury due to peritubular capillaries damage, and the said levels inversely correlate with renal function (estimated glomerular filtration, eGFR) after medical treatment in essential and renovascular hypertensive patients [117].

Intensive treatment of T2DM patients suffering an acute coronary attack showed decreased endothelial CD31+/CD41+ EVs levels [118]. Administration of pioglitazone to patients with metabolic syndrome reduced endothelial EV levels [119]. In patients with T2DM and hypertension, endothelial EV levels correlate directly with the mean systolic and pulse blood pressure but inversely with eGFR compared with normotensive diabetic patients [120]. CD31+/CD42− [121,122] and CD31+/CD42−/CD51+ [123] endothelial-derived EVs are increased in hypertensive patients with T2DM correlating these levels with mean arterial pressure and mean systolic blood pressure.

From the studies explained above, endothelial-derived EVs can be considered an endothelial damage marker. In addition, along with EVs secreted from other sources such as platelets and leukocytes, endothelial-derived EVs play an active role in the pathogenesis of hypertension. Increased levels of EVs relate to a smaller ability of vessels to regenerate, increasing cardiovascular risk and nephropathy [124]. All these studies point to the importance of assessing plasma EV levels to establish the risk of organ damage in diabetes.

In a different approach, EVs would have beneficial effects as carriers of signals to preserve, for example, endothelial function and vessel integrity in vascular diseases [125,126]. Indeed, EVs have therapeutic potential as vehicles for transferring and secreting different molecules (cytokines, chemokines, growth factors, nucleic acids, etc.) to other targets in disease [127]. Furthermore, EVs from mesenchymal stem cells can preserve myocardial function after ischemia/reperfusion in animal models and humans [128,129,130]. Moreover, EVs derived from bone marrow CD34+, or endothelial progenitor cells, increase cardiac viability by decreasing oxidative stress and activating PI3K/Akt pathway and promoting angiogenesis [131,132,133]. In addition, EVs derived from cardiac progenitor cells protect the myocardium from ischemia/reperfusion injury [134].

EVs have advantages in regenerative medicine and therapy because they maintain their properties during long storage periods. Thus, the limitations of using viable cells that can undergo aberrant differentiation are avoided.

EVs could be useful vectors in gene therapy by transporting and delivering nucleic acids. For instance, PI3K/Akt pathway mRNAs carried by endothelial progenitor cells-derived EVs promote angiogenesis response in endothelial cells after EVs and endothelial cell fusion [135]. Circulating EVs also carry miRNAs known for their implication in the pathophysiology of cardiovascular and other diseases by modulating target cell gene expression [136], and specific miRNAs are expressed and packed in circulating EVs in these diseases [137]. This compartmentalization is stimulus-dependent. This is similar to hypoxia which determines the regenerative properties of mesenchymal stem cells-derived EVs and the expression of pro-angiogenic miRNAs in endothelial progenitor cells-derived EVs [128,138,139]. It has been demonstrated that miR-126 is carried by circulating EVs for regulating angiogenesis and vascular integrity [108,140,141]. miR-126 transported into recipient human coronary artery endothelial cells by endothelial EVs released by apoptotic endothelial cells promoted reendothelialization. Still, hyperglycemia lowered the amounts of miR-126 transported and reduced endothelial repair capacity in vivo [141]. Interestingly, patients with coronary artery disease have low levels or lack miR-126 compared with healthy subjects [138,139], indicating the importance of EVs cargo in developing and treating the disease.

## 3. Unraveling Underlying Mechanisms: Therapeutical Approaches

As mentioned previously, aging is a significant risk factor for many human diseases, especially in CRS, a pathology considered an age-related chronic disease.

A gradual decline in physical and cognitive function during the aging process leads to a higher risk of illness. The World Health Organization (WHO) indicated that age-related diseases have increased in the last century due to the increase in lifespan and predicts a doubling of the world’s population aged over 60 years by 2050 [142]. Therefore, improving the quality of health, nutrition, education, income, and medicine are strategic actions to delay aging and age-related diseases [143] and research efforts to understand the biological mechanisms underpinning age-related chronic diseases are vital. Nonetheless, increased vulnerability in premature aging, CKD-associated CVD triggers pathophysiological processes such as chronic inflammation, immune activation, dysregulation of the musculoskeletal and endocrine systems, oxidative stress, energy imbalance, endurance, are briefly characterized by a reduced physiological function, which can lead to frailty [144,145]. However, even though in CKD, the biological mechanism that causes frailty is unknown, the frailty in CKD patients may be due to CVD comorbidities.

Moreover, chronic systemic inflammatory state characteristic of frail patients is also found in advanced CKD patients [146], especially those in renal replacement therapy [147]. At the beginning of the 20th century, the prevalence of frailty in the elderly population was 11% compared with 60% in HD patients [145]. Thus, the main problem of aging or age-associated diseases such as CRS is frailty aggravated by consistent and low-grade systemic inflammation environments. In this case, cells lose resilience against external injuries and are close to acquiring senescent phenotype; therefore, senescence is intimately associated with frailty [148]. Recently, Boccardi and Mecocci highlighted the role of cellular senescence with advanced age-related CVD and frailty [149].

This idea is not accurate because, in contrast with the preconceived frailty concept, the loss of cellular resilience is not associated with pathology or aging. A far as we know, it is of note that the number of frail patients reaching end-stage kidney disease is increasing [145]. In addition, frailty has been associated with an increased risk of CVD [27,150]. However, patients who reverse the frailty state also prevent the development of CVD [144].

In contrast, acquiring senescent phenotype by aging or age-related chronic disease, among others CKD-associated CVD, is an irreversible pathophysiological process [22,53,151]. Therefore, some therapeutic approaches emerged focusing on eliminating senescent cells using compounds called senolytics [151]. Accordingly, senomorphics are drugs that can delay the appearance of senescent cells or can inhibit the senescent cell detrimental effects [151]. More recently, other therapeutic drugs have been developed to modulate the proinflammatory senescent secretome (senostatics) [152]. Each of these therapies appear to be helpful to delay aging and age-related diseases [152,153].

More importantly, in the cell–cell communication system during senescence development, EVs act as regulators of several physiological processes and participate in the development and progression of multiple diseases, including EVs delivered from senescent cells in pathologies associated with premature aging such as CRS [154,155].

Cells acquire a senescent phenotype due to the multifactorial causes of aging and age-related diseases, and therefore some therapeutic approaches have been developed to delay the accumulation and/or eliminate senescent cells. To date, the most important treatments are antioxidants [156], senolytics, senomorphics, and senostatics [152,157], and the intervention in senescence cell-associated EVs that serve as therapeutic targets and tools [158]. Thus, studies related to the senescence field are essential for developing drugs that can eliminate senescent cells. In addition, the modulation of intercellular communication could also have a therapeutic potential to treat age-related diseases and CVD and/or CRS.

### 3.1. Senolytics, Senomorphics, and Senostatics

In recent years, several studies have focused on designing and examining the potential of selective drugs to delay premature aging associated with chronic inflammatory pathologies, especially CRS, to decrease senescent cells’ accumulation in several tissue and organs in aging. The main objective is to stop the harmful effects of senescent cells in the evolution of chronic diseases.

Much effort has been recently made to therapeutically target detrimental effects of cellular senescence, including selectively killing senescent cells (senolytics), delaying the senescence-phenotype (senomorphics) [157], and modulating a proinflammatory senescent secretome (senostatics) [152]. Whereas senolytics are drugs that can be dead cells that target selectively senescent cells, senomorphics can modulate the secretory phenotype of senescent cells. Therefore, these agents can delay or stop the senescence process. Senostatics are drugs that slow or stop the process in the same way as senomorphics; their target is the pro-inflammatory cytokines released by senescent cells. Thus, the clearance of senescence cells through these drugs appears promising for the treatment of age-related diseases such as CKD or CRS [152,153].

**Figure 2:**
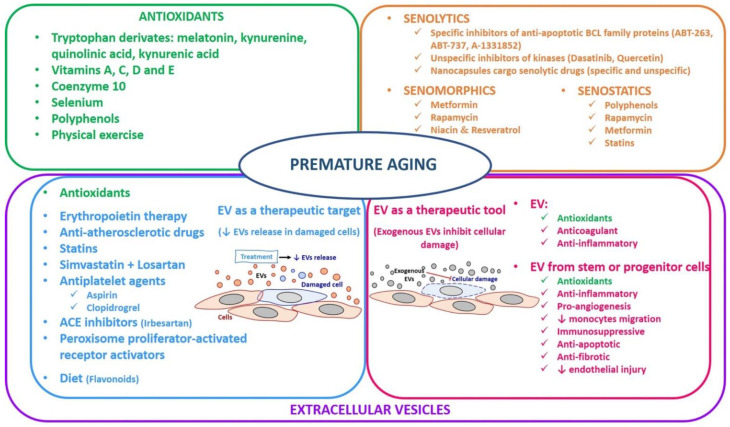
Therapeutical approaches in premature aging.

In the case of senolytic drugs, they mainly target proteins involved in apoptosis, such as B-cell lymphoma 2 (Bcl-2) family members, phosphoinositol 3 kinase/protein kinase B (PI3K)/AKT), and fork head box transcription factor-p53 (FOX04-p53) axis. These agents induce the senescent cells’ apoptosis selectively. In this regard, some chemical compounds could have a senolytic effect, as shown in Figure 2: (1) specific inhibitors of anti-apoptotic BCL family proteins (ABT-263 or Navitoclax, ABT-737, A-1331852) and (2) unspecific inhibitors of kinases (Dasatinib, Quercetin), which cannot distinguish between senescent and normal cells, could therefore be associated with several undesired side-effects [157,159]. Recently, a new approach has been developed using nanocapsules whose cargo are senolytic drugs (specific and unspecific) in mice to deliver to the senescent cells [157]. In this regard, senotherapy was used to treat senescent cells’ accumulation in CVD, preventing disease evolution [160]. In this sense, preclinical studies have focused on preventing or reversing a wide range of aging and premature aging diseases, CVD associated-CKD, using senolytic drugs [160]. However, the field is still new, and before administrating these drugs to humans, clinical trials shall be conducted.

Another approach is senomorphics that delay SASP also referred to as SASP inhibitors. In this case, SASP is characterized by a secretory phenotype between cytokines, chemokines, and growth factors that mediate paracrine and autocrine signaling in the development of senescence [20]. In this way, senomorphic drugs modulate the SASP and stimulate the immune system to clear the senescent cells [161]. The target of the senomorphics are kinases, pro-inflammatory mediators, mammalian target of rapamycin (mTOR), and PI3K/AKT [157]. The main disadvantage of senomorphics is their unspecificity for senescent cells. Nevertheless, some therapeutical drugs have been shown to modulate SASP in CKD [161] (Figure 2):

- Metformin (used to treat type 2 diabetes mellitus): presents a role in diabetic nephropathy because it attenuates age-related diseases through Nuclear Factor Kappa B NF-κB inhibition [162].
- Rapamycin (used as an immunosuppressor after organ transplant): treatment with rapamycin delays death in an in vivo fibrotic kidney model of mice [163].
- Niacin and resveratrol activate sirtuin and inhibit NF-κB signaling, which is altered in reduced kidney function [164,165].

Finally, the senostatic approach prevents the progression of senescence, modulating the senescent inflammation (Figure 2). Senotatics’ role is very similar to senomorphics because they inhibit SASP indirectly. Remarkably, polyphenols with their antioxidant and anti-inflammatory properties have been considered senostatics. Interestingly, resveratrol could also be considered senostatic due to the fact that it inhibits senescent cells in cardiovascular complications [166]. Moreover, some authors believed that senostatics such as rapamycin, metformin, and statins had been shown to mitigate the pathological cell senescence associated with atherosclerosis and CVD in humans [167].

In general, various therapeutic approaches, including senolytics, senomorphics, and senostatics, have emerged as a strategy to mitigate/alleviate age-related diseases, among them, CRS.

### 3.2. Antioxidants

In cardiac and renal disorder or CRS, CVD is the leading cause of death in CKD patients [81]. ESRD is a terminal illness characterized by a high reduction of kidney function and appears when the glomerular filtration rate is less than 15 mL/min. These patients have been treated with dialysis. Patients in HD are the maximum exponent of the oxidative stress and inflammatory situation in which the clinical evolution is fast. CVD development in uremic patients involves complex oxidative stress, inflammation, and endothelial dysfunction processes, resulting in CVD, such as atherosclerosis. Both oxidation and inflammation increase for different reasons in HD, with uremic toxins playing a decisive role [40]. Moreover, CKD-related pathologies increase ROS generation and, on the other hand, are associated with a defect in antioxidant machinery, both resulting in an imbalance and accumulation of oxidative stress in the organism [81].

There is some evidence regarding the relationship of other uremic toxins (such as p-cresol and indoxyl sulfate) with oxidative stress and inflammation. For example, it has been demonstrated that ROS increases the Nuclear Factor Kappa-B (NF-κB transcription factor), which regulates the synthesis of the proinflammatory cytokine [81,168]. Furthermore, the increment of proinflammatory factors stimulates the immune system and kidney filtration failure [169].

Some antioxidant compounds have been administered in CKD patients to prevent illness related to CRS and its progression. Nowadays, melatonin, a tryptophan derivate, appears in the list of uremic toxins described to date, although its role in CKD is unknown, and its main role is as an antioxidant [138]. Moreover, to date, it has been shown that exogenous melatonin administration inhibits oxidative stress in vivo [170]. Moreover, aging is associated with increased ROS and a reduction of endogenous melatonin secretion [171].

Other tryptophan metabolites such as kynurenine, quinolinic acid, and kynurenic acid are increased in CKD patients and play a key role in generating oxidative stress in CKD. Moreover, these levels are associated with increased antioxidant enzymes and the prevalence of CVD in patients with end-stage renal disease [170,172].

Recently, due to the important role of oxidative stress in the pathogenesis of aging and premature aging diseases such as CRS, some studies have been conducted to investigate the therapeutic approach of the antioxidants [5,173] (Figure 2):

- Vitamins A, C, and E: the higher intake of these vitamins lowers CVD risk and type 2 diabetes mellitus [173,174].
- Vitamin D: its deficiency is characteristic in CKD patients. This vitamin is important in redox balance, endothelial function, and immunity. Moreover, vitamin D disorder is associated with calcium phosphate disbalance and increased oxidative stress in the pathogenesis of CKD [81]. For this reason, CKD patients are recommended to take calcitriol.
- Coenzyme Q10: plays a role in the mitochondrial respiratory chain, and therefore, oral administration is an antioxidant strategy in chronic pathologies associated with mitochondrial dysfunction [175].
- Selenium: is involved in oxidative stress because some antioxidant enzymes are selenoproteins. This element is essential to prevent inflammatory diseases, CVD, diabetes mellitus, stroke, CKD, and cancer [176].
- Polyphenols: are derivatives from fruits, vegetables, and cereals. Quercetin and resveratrol are present in red wine. Both act as antioxidants that prevent diseases such as CVD, hypertension, diabetes mellitus, and cancer. Although this has not been firmly established, they are known for their antioxidant and anti-inflammatory properties [173,177].
- Physical exercise: aging and/or physical inactivity/sedentary lifestyle increase oxidative stress, especially in skeletal muscle. A healthy, active lifestyle and regular and moderate exercise are critical to maintaining an optimal state of health due to reduced oxidative stress, and therefore, it is beneficial to prevent chronic diseases [178].

In general, it is highlighted that antioxidants therapies, vitamins, ions, polyphenols, and physical exercise reduce the oxidative stress levels that used to be associated with aging and premature aging, such as CRS. Moreover, these antioxidant therapies have been shown to reduce the frailty incidence, but there is no lifespan extension [179].

However, clinical trials involving antioxidant supplementation in the treatment of several aging-associated diseases often show conflicting results and lead to dangerous misconceptions. Firstly, the linear dose-response relationship between increasing amounts of ROS and biological damages is currently being replaced by a modernized view of this theory that considers the so-called “mitohormesis” (a biological response where the induction of a reduced amount of mitochondrial stress leads to an increment in health and viability within a cell, tissue, or organism). Secondly, the genetic background of the patients enrolled in the studies should also be considered for the conflicting results. This is because longevity depends not only on lifestyle habits but also on genetic background. Lastly, controversies might also be due to many aspects, among which the often-limited statistic power of the studies. The patient initial quantitative redox state, the bioavailability of the molecules used, the non-specific effects that antioxidants might have in the human body, and the validity of the biomarkers used to determine the effects of antioxidants on human health should be taken into account [173].

Therefore, based on the factors mentioned above, the effect of the antioxidant therapies should be re-evaluated and considered as a preventive therapy for aging and premature aging-related diseases.

### 3.3. Extracellular Vesicles

Since the discovery that EVs can transfer biological information and mediate beneficial effects, their use as drug delivery tool vehicles has gained scientific interest [180]. This highlights that EVs may serve as diagnostic and therapeutic targets and tools [98]. Therefore, in this section, we focus on discussing the role of EVs in the initiation and evolution of chronic inflammatory diseases (EVs as a biomarker in the clinical diagnosis) and the recent advances in EVs as a therapeutic target and therapeutic tool (Figure 2).

#### 3.3.1. Extracellular Vesicles in Clinical Prognosis/Diagnosis as a Biomarker

EVs can be used as a clinical diagnostic biomarker in biological function, pathogenic procedures, and pharmacological response; therefore, EV characterization, quantification and biological cargo could be used by therapeutic intervention. There are some advantages: (1) EV assessment is an analytical tool to quickly measure and evaluate their level in blood or plasma, (2) These data are helpful to assess the risk or identify pathologies. The disadvantages are: (1) EV evaluation requires blood extraction, which is an invasive technique, and (2) EV parameter measurement could be expensive. Interestingly, EV evaluation makes it possible to identify individuals with high pathological risk, diagnose diseases, and treat patients [181,182,183]. In addition, EVs allow early detection and carry out a therapeutic intervention before the disease progresses irreversibly or worsens in atherosclerosis [181], in kidney diseases [181,183,184] and CRS [184].

We have represented in Table 1 that EVs participate in the etiopathogenesis of multiple CVDs, particularly in the onset of kidney diseases [185]. Therefore, there is great interest in evaluating the changes in EV levels in response to drug treatment [26,186]. On the one hand, there is the possibility of acting at the production and release of EVs. On the other hand, current difficulties which influence both processes should always be considered because the cellular mechanisms involved are not completely clear [26,186].

#### 3.3.2. Extracellular Vesicles as a Therapeutic Target (Therapeutical Approach)

Different studies have shown that specific pharmacological treatments targeting EVs decreased their levels in CVDs [28,187,188]. Therefore, the premature aging associated with these chronic inflammatory pathologies highlighted CVD-associated-CKD [28,189].

During CKD progression due to the accumulation of uremic toxins, EVs generated from different cell types induce endothelial dysfunction because they are responsible for increasing oxidative stress, reducing the bioavailability of nitric oxide, and producing chronic cardiovascular inflammation [26,190]. Knowledge regarding their formation and release represents an attractive therapeutic target to limit EV levels, but the release mechanisms are not fully elucidated. As far as we know, direct or indirect inhibition of EV generation and/or liberation is a more effective proposal in CKD and other inflammatory diseases [26,191].

The regulation of EVs release on plasma, or drug uptake by target cells, reduces cardiovascular risk in inflammatory diseases, including CKD. Furthermore, these drugs could mediate a reduction in EV concentrations in plasma, having a beneficial effect on the etiopathogenesis and the evolution of chronic diseases [26,187,191]. Some of these drugs are described in Table 2.

Moreover, different authors have highlighted the importance of diet on the release of EVs, perhaps these being one of the mechanisms involved in the role of diet in the development of cardiovascular pathologies [191,195]. In the case of flavonoids, they improve endothelial function as they decrease the levels of endothelial EVs [181].

Another factor to consider is that some drug treatments and pathologies and their comorbidities may change the biosynthesis and release of EVs, therefore, modifying their capacity of interaction with the target cells and their subsequent effect in the subject [196].

#### 3.3.3. Extracellular Vesicles as a Therapeutic Tool

EVs have been studied as a therapeutic tool to delay or treat many pathologies in the last years. In this context, EV phenotypes and their origin, source, or parental cell are critical due to their role in modulating cellular processes and mechanisms. The main reason is that the cargo of EVs could be similar to the cell that generated it and depend on their features to induce tissue repair after reprogramming the target cell [197]. Furthermore, all the EVs contain various biomolecules with some properties: anticoagulant, anti-inflammatory [198], and antioxidants [195,199,200]. Moreover, recent studies showed the beneficial effects of EVs from the stem or progenitor cells in chronic inflammatory diseases which are associated with premature aging (Table 3 and Table 4).

In general, the limitations of EVs as a therapeutic tool are (1) to obtain enough EVs, which depend on the methods of EVs production and isolation, and (2) human therapy requires a high number of EVs. In contrast, due to the ability of EVs to overcome natural barriers, their cell communication properties, and their circulation stability, EVs can provide multiple advantages as a drug delivery system currently available for targeted therapies.

#### 3.3.4. Beneficial and Preventive Effects of Physical Activity and Diet in Cardiovascular and Renal Diseases Mediated by EVs

Lifestyle interventions, such as diet and exercise, have benefits for healthy and diseased people, for instance increasing lifespan and avoiding or delaying the onset of many diseases [208,209,210] such as CVD. In this context, circulating EVs emerged as a signaling mechanism to spread those benefits affecting many cell functions.

Concerning regular physical activity, it has been proven that it has benefits for healthy and ill people, such as an increased lifespan and the avoidance or delay of diseases even when the physical activity is started late in life [211,212,213]. Exercise does not only prevent the onset of obesity, T2DM, CVDs (typically hypertension), Alzheimer’s, anxiety, depression, fibromyalgia, rheumatoid arthritis, osteoporosis, bone, muscle, and joint disorders but also helps in the pharmacological treatment of these pathologies [214]. The benefits of exercise affect all organs using a complex network of cytokines and messengers released by different organs [215]. As circulating EVs carry signaling molecules or genetic material throughout the body, they are an excellent mechanism to spread exercise-induced changes.

An increasing number of studies demonstrated changes in the profile of EVs after exercise depending on the intensity and kind of physical activity performed [216,217,218,219,220,221]. One explanation for the positive effect of physical activity on health and disease is the regulation of oxidative stress by EVs. miR-146 content in endothelial-derived EVs increased with high-intensity interval aerobic exercise and endurance training [222]. miR-146 reduces NADPH oxidase 4 (NOX4) expression, ROS generation, and inflammation in endothelial cells [223,224]. Thirty-minute cycling at 70% VO_2_ peak for 8 weeks, increasing the intensity over time, increases nuclear factor erythroid-2-related factor 2 (Nrf2) responses in young and old participants [225]. Nrf2, a transcription factor, is a central regulator in oxidative stress conditions. miR-93 and miR-145-5p reduce Nrf2 protein content in all tissues, and exercise decreases the EVs carrying them [226]. EVs can improve antioxidant and detoxifying gene expression depending on their levels.

Moreover, exercise modulates immunosenescence and inflammaging through the regulation of acetylcholinesterase activity, reducing the proinflammatory effect of Acetylcholine [227,228,229]. Remarkably, circulating exosomal miRNA profile showed cholinesterase-targeting miRNAs identified in silico, specifically miR-148a, miR-16-2-3p, miR-28-5p, miR-203-3p, and miR-218-5p, at baseline in endurance-trained elderly men, and miR-218-5p increased immediately after a single bout in sedentary older men [230].

It was also proposed that aerobic exercise modulate aging and inflammation by modulating circulating cytokines such as IL-1β. Aerobic exercise increased circulating levels of EVs carrying IL-1β and decreased circulating free cytokine in experimental animals preventing the proinflammatory action in aged mice [231]. The mechanisms described above could explain the effect of aerobic exercise in humans.

As physical training prevents the development of obesity, huge efforts are being made to find the drug to terminate it. Regular physical activity is a lifestyle strategy to improve the quality of life and health of overweight people. As occurs with other pathologies, there can be a role for EVs in obesity. High-intensity interval aerobic exercise increases some miRNAs in EVs in overweight and normal-weight women [222]. It is known that one of these miRNAs is increased in adipose tissue of high-fat diet animals [232], so its clearance in EVs could prevent its effect in different tissues. miR-223-3p in circulating EVs increased in acute and chronic exercise in obese and aged subjects [222,230]. miR-233 targets are involved in developing obesity and T2DM [233]; therefore, improved clearance through EVs by exercise avoids the development of these pathologies. Authors consider miR-233 in circulating EVs a biomarker of obesity and an index of therapeutic responses or an indicator of exercise efficacy in overweight and obese groups, including the elderly [234].

T2DM patients obtained benefits of physical training too. T2DM patients with coronary artery disease, albuminuria, and microalbuminuria had higher levels of circulating endothelial related EVs, monocyte-derived EVs, platelet-derived Evs, and EVs from endothelial progenitor cells (EPCs) [235]. Increased circulating EVs from endothelial cells indicate vascular injury progression, atherosclerosis, and nephropathy [124,236,237,238]. Higher levels of platelet EVs indicate activation and risk of atherothrombosis and cardiovascular events [239,240,241]. Increased monocyte-derived EVs augment the risk of atherothrombosis, glomerular inflammation, and an increase of permeability and microvascular damage [242,243]. EVs derived from endothelial progenitor cells carry miRNAs involved in vascular repair [244]; therefore, the increasing levels indicate vascular damage. When patients follow 12 months of the aerobic resistance training program, endothelial progenitor cells derived circulating EVs are significantly increased, indicating a beneficial effect of exercise in the vascular endothelium. In addition, EVs with procoagulant effects decreased. In conclusion, the increased circulating EVs in T2DM with albuminuria and coronary artery disease is a marker of disease severity, and regular exercise has some beneficial effects in these patients.

Circulating EVs can also mediate the beneficial effects of regular physical activity in CVDs. For instance, miR-21 levels carried inside EVs increased with exercise [245]. miR-21 have many cardioprotective effects: favors a reparative and angiogenic macrophage phenotype in the infarct zone [246], inhibits cell apoptosis [247], increases nitric oxide synthase activity [208], and promotes angiogenesis by increasing expression of hypoxia-inducible factor-1 (HIF-1α) and of vascular endothelial growth factor (VEGF) and activating PTEN/AKT signaling [209].

Circulating EVs represent a cross-talking mechanism between cells in CVDs. For instance, miR-342-5p carried by endothelial EVs is released and internalized by cardiomyocytes inhibiting JNK2 apoptotic signal and increasing viability after hypoxia and reoxygenation [221]. Ischemic cultured cardiomyocytes secrete EVs carrying miR-122 and miR-143, which promote angiogenesis in vivo [210]. miR-122 is upregulated in endurance-trained healthy men and women [248], which is remarkable because it could bring to light an exercise-dependent angiogenic role in humans. Moreover, EVs obtained from trained coronary syndrome patients stimulated reendothelialization using aortic human endothelial cells in culture [249].

Regarding other physiological interventions, diet is a lifestyle intervention that potentially prevents many diseases. Similar to exercise, diet can have beneficial effects altering EVs shedding, contents, and levels.

For instance, in the PREDIMED study, intake of a Mediterranean diet complemented with nuts or extra-virgin olive oil reduced more prothrombotic EVs shedding from vascular and blood cells in patients with high cardiovascular risk compared with a low-fat diet. Therefore, the Mediterranean diet is more effective to decrease atherothrombosis in these individuals [250].

On the other hand, polyphenols from berries, absorbed in the intestine and detected in blood [251], can decrease oxidative stress [252,253,254] and inflammation [255,256], increase NO production [257] and improve lipid profile [256,258]. Due to those effects, dietary berries are considered beneficial for preventing CVDs [259]. A group of patients with myocardial infarction supplemented with bilberry extract every day for 8 weeks reduced endothelial and platelet vesiculation and vesicle gene transcription [260]. A mixture of berries extracts decreases platelet aggregation and granule secretion [261,262,263]. In addition, anthocyanins, entering endothelial cells from plasma, decreased vesiculation, oxidative stress, and inflammatory and procoagulant state activation [264,265,266,267].

In summary, changes in circulating EVs cargo could be one of the mechanisms by which exercise produces beneficial effects in health and disease at any age. Therefore, deep knowledge about these changes could be used as biomarkers of efficacy and exercise recommendation. In addition, the ability of diet and some components of food-derived molecules to avoid the increase of EV levels involved in atherosclerosis and other CVDs could explain their preventive effect.

## 4. Conclusions

Oxidative stress, senescence, and inflammation are related to aging. As a result, aging is accompanied by an increased prevalence of age-related chronic diseases, and one of the most prevalent are CVDs, including hypertension, atherosclerosis, and heart failure. In addition, oxidative stress, senescent cells accumulation, and the chronic inflammatory process increase the susceptibility to these diseases in the elderly. Furthermore, due to the growth of the elderly population and the increase of other risk factors such as diabetes mellitus, obesity is expected that age-related chronic increase by 10% over the next 20 years [30,32]. Therefore, it seems necessary to improve strategic actions to delay aging and age-related diseases and increase the research efforts to understand the biological mechanisms underpinning age-related chronic diseases. In this regard, it has been suggested that EVs could be both novel clinical biomarkers and new therapeutic targets for age-related diseases. Despite the fact that their characterization and classification are still updating, their cargo and their origin may be helpful as specific biomarkers in pathologies associated with aging such as CRS. Moreover, recent studies have shown that EVs could be applied as a therapeutic tool to inhibit or delay the development of age-related chronic diseases.

## Figures and Tables

**Table 1:** Principal features of EVs.

General Characteristics of EVs			
Types of EVs	Different Classification by	Process Mediated	EVs Used as
Exosomes	Size	Physiological/homeostasis (beneficial effects)	Biomarker (in clinical prognosis or diagnosis)
Microvesicles/Microparticles	Morphology	Pathological effect: chronic inflammatory diseases (CKD, diabetes, hypertension, peripheral vascular disease, heart failure, and CKD).	Therapeutic target
Apoptotic bodies	Biochemical composition		Therapeutic tool
Small or large EVs	Release mechanism		

**Table 2:** EVs are involved in the pathogenesis of CVD-associated-CKD.

	EVs as Clinical Prognosis/Diagnostic Biomarker in Chronic Diseases				
Origin (Cells)	Species	Levels (Plasma) ↑: Increase ↓: Decrease	Diseases	Effect	References
Leukocytes Platelets Endothelial	Human	↑	Initiation and progression of CVD (associated with inflammation)	Apoptosis/activation platelets, leukocytes erythrocytes, and endothelial cells Endothelial function and angiogenesis Inflammation and thrombosis	[21,187]
Leukocytes Platelets Endothelial	Human	↑	Atherosclerosis (chronic inflammatory)	Vascular injury Inflammation Pro-thrombotic state	[21]
Endothelial Blood	Human	↑	CKD (including cardiovascular events)	Chronic inflammation	[26]
Platelets Erythrocytes Endothelial	Human	↑	Metabolic syndrome (inflammatory diseases)	Metabolic complications Vascular effects Immuno-inflammatory responses	[192]
Platelets Endothelial	Human	↑	Type 1 Diabetes Mellitus	Inflammation Autoimmunity	[193]
Monocyte subpopulations	Human	↑	CKD on HD (mainly people with diabetes)	Progression of the CVD in patients with CKD CKD in HD patients with DM	[101]
Senescent endothelial cells from plasma elderly subjects	Human (in vivo and in vitro)	↑	Vascular calcification	Marker for atherosclerosis Premature vascular disease associated with CKD	[20]
From indoxyl-sulfate treated endothelial cells (studies in vitro)	Human (in vitro)	↑ (from culture supernatant)	Vascular calcification in CVD associated-CKD	Modulation of pro-inflammatory genes in VSMCs Modulation of mediators involved in calcification progression in VSMCs	[189]
Endothelial	Human	↑	CKD	Vascular inflammation (acute or chronic) Endothelial dysfunction	[194]

**Table 3:** EVs as target (pharmacological modulation of plasma EVs).

	Extracellular Vesicles as a Therapeutic Target (Therapeutical Approach)				
Drugs	Species	EVs Levels (Plasma) ↑: Increase ↓: Decrease	Diseases	Beneficial Effect	References
Antioxidants	Human	↓	Inflammatory pathologies: atherosclerosis, CKD, CVD, CVD associated-CKD Hemostasia disorders Aging	Improved endothelial function ↓ evolution of chronic disease (CVD associated-CKD)	[158,185,195]
Antioxidants	Human	↓	Atherosclerosis Diabetic patients Dyslipidaemic patients	↓ endothelial injury ↓ platelet activation	[187]
Erythropoietin therapy	Human	↓ (endothelial EVs)	CKD in the end-stage	↓ shear stress	[201]
Anti-atherosclerotic drugs (angiotensin-II receptor antagonists or blockers)	Human	↓	Hypertension patients	↓ endothelial injury ↓ coagulation ↓ inflammation	[196,202]
Statins	Human	↓	CVD (the process of atherogenesis)	↓ cholesterol ↓ vascular inflammation ↓ platelet aggregation	[187,203]
Simvastatin + Losartan	Human	↓ (monocyte-, endothelial- and platelet-EVs)	Patients with hypertension Patients with type 2 diabetes	↓ cholesterol ↓ endothelial injury ↓ coagulation ↓ inflammation	[187]
Peroxisome proliferator-activated receptor (PPAR) activators	Human	↓ (platelet-derived EVs)	DyslipidaemiaType 2 diabetes	Anti-inflammatory properties	[187]
Antiplatelet drugs (Aspirin, Clopidogrel)	Human	↓ (platelet- and endothelial-derived EVs)	Coronary disease	↓ platelet aggregation	[187,202]
Angiotensin-converting enzyme (ACE) inhibitors (Irbesartan)	Human	↓	atherosclerosis	↑ endothelial progenitor cells	[181]

**Table 4:** EVs as treatment (therapeutical tool).

Extracellular Vesicles as a Therapeutic Tool				
EVs Type	EVs Levels ↑: Increase ↓: Decrease	Effect	Features	References
Platelet	↑	↑ endothelial progenitor cells	Vascular endothelial repair	[98]
Endothelial	↑	Protein C activation (↓ thrombin and ↓ tissue factor)	Anticoagulant	[198]
Endothelial	↑	↓ cytokine expression (IL-6 and TNF-α)	Anti-inflammatory	[198,204]
**Extracellular Vesicles from the Stem or Progenitor Cells as a Therapeutic Tool**				
**Treatment**		**Model**	**Effect/Properties**	**References**
EVs-Mesenchymal Stem Cell (MSC)		cardiovascular model in vitro (Inflammatory endothelial damage)	↓ endothelial injury Anti-inflammatory Pro-angiogenesis ↓ monocytes’ migration Immunosuppressive	[197,205]
EVs-MSC		Acute kidney injury in mice	Anti-apoptotic feature	[197]
EVs-MSC		Rat model chronic liver fibrosis	Anti-fibrotic Anti-inflammatory	[197]
EVs from a different stem cell (specially MSC-EVs)		Acute kidney injury (AKI) CKD	↓ inflammatory response ↓ Fibrosis ↓ oxidative stress ↓ cell death	[206]
EVs-Adipose derived stroma cell (ADSC)		CRS	↓ cardiac fibrosis	[207]
EVs from multiple origins		CKD	Antioxidant effect in kidney diseases	[180]

## Abbreviations

ADSC: adipose derived stromal cell; AKT: protein kinase B; Bcl-2: B-cell lymphoma 2; CKDs: chronic kidney diseases; CRS: cardiorenal syndrome; CVDs: cardiovascular diseases; eNOS: endothelial nitric oxide synthase; EPC: endothelial progenitor cells; ESRD: end stage renal disease; EVs: extracellular vesicles; FOX04: fork head box transcription factor 4; GFR: glomerular filtration rate; GPx: glutathione peroxidase; HD: hemodialysis; HFpEF: Heart failure with preserved ejection fraction; HIF-1α: hypoxia inducible factor-1 alpha; ICAM-1: intercellular adhesion molecule-1; IL-1: interleukin-1; IL-6: interleukin-6; IL-8: interleukin-8; iNOS: inducible nitric oxide synthase; JNK2: c-Jun N-terminal kinase 2; LV: Left ventricular; MCP-1: monocyte chemoattractant protein-1; MSC: mesenchymal stem cell; mTOR: mammalian target of rapamycin; NF-κB: transcription factor Nuclear Factor Kappa-B; NO: nitric oxide; NOX4: NAPDH oxidase 4; Nrf2: nuclear factor erythroid-2-related factor 2; O_2_^•−^: superoxide anion; ONOO^−^: peroxynitrite; ox-LDL: oxidized-low density lipoproteins; PI3K: phosphoinositol 3 kinase; PTEN: phosphatase and tensin homolog; RONS: reactive oxygen and nitrogen species; SASP: senescence-associated-secretory phenotype; SOD: superoxide dismutase; T2DM: type 2 diabetes mellitus; TNF-α: Tumor necrosis factor alpha; VCAM-1: vascular adhesion molecule-1; VEGF: vascular endotelial growth factor; VSMCs: vascular smooth muscle cells.
